# Clear Cell Carcinoma of Minor Salivary Glands: The Nasopharynx, an Uncommon Site of Origin

**DOI:** 10.7759/cureus.25033

**Published:** 2022-05-16

**Authors:** Samia Arifi, Nawal Hammas, Mohamed Ait Erraisse

**Affiliations:** 1 Medical Oncology Department, Hassan II University Hospital, Faculty of Medicine and Pharmacy, Sidi Mohamed Ben Abdellah University, Fez, MAR; 2 Pathology Department, Hassan II University Hospital, Faculty of Medicine and Pharmacy, Sidi Mohamed Ben Abdellah University, Fez, MAR; 3 Radiation Oncology Department, Hassan II University Hospital, Fez, MAR

**Keywords:** radiotherapy, chemotherapy, nasopharynx, minor salivary glands, clear cell carcinoma

## Abstract

Clear cell carcinoma is a rare minor salivary gland neoplasm. Its occurrence in the nasopharynx is uncommon. A limited number of cases are reported in the literature. Here, we report an additional case of clear cell carcinoma of the nasopharynx managed by induction chemotherapy followed by chemoradiotherapy, and we describe the clinical presentation, pathological features, and outcome.

A 63-year-old man presented with an exophytic, ulcerative, and easily hemorrhagic tumor on the left side of the nasopharynx. A diagnosis of primary, cT4N0M0, clear cell carcinoma of the minor salivary gland was confirmed by a core needle biopsy through nasopharyngoscopy and staging procedures. The patient was treated by induction chemotherapy followed by concomitant chemoradiotherapy with clinical benefit and disease stabilization.

Primary salivary gland clear cell carcinoma of the nasopharynx is uncommon. A definitive diagnosis requires an appropriate workup. The optimal treatment is unclear. Chemoradiotherapy might be a good option to manage such cases.

## Introduction

Minor salivary gland tumors are uncommon malignancies, representing about 2% of all head and neck cancers [1]. Primary tumor locations correspond to the anatomic distribution of minor salivary glands throughout the aerodigestive tract. The site of origin is mainly related to the density of minor salivary glands in a particular tissue [1]. The most frequent primary sites are the oral cavity, nasal cavity, and paranasal sinuses, and they rarely arise from the nasopharynx [1]. According to the World Health Organization (WHO) classification [2], tumors of minor salivary glands comprise different histological types. Clear cell carcinoma, not otherwise specified, is an extremely rare entity, accounting for nearly 1% of all minor salivary gland tumors [3]. They are considered low-grade tumors [2]. The major treatment of minor salivary gland tumors is surgery [1]. Data on the efficacy of chemoradiotherapy are limited [4]. Here, we report a rare case of clear cell carcinoma of the nasopharynx managed by chemoradiotherapy. The clinical presentation, pathological features, therapeutic procedures, and outcome are described.

## Case presentation

**Figure 1:**
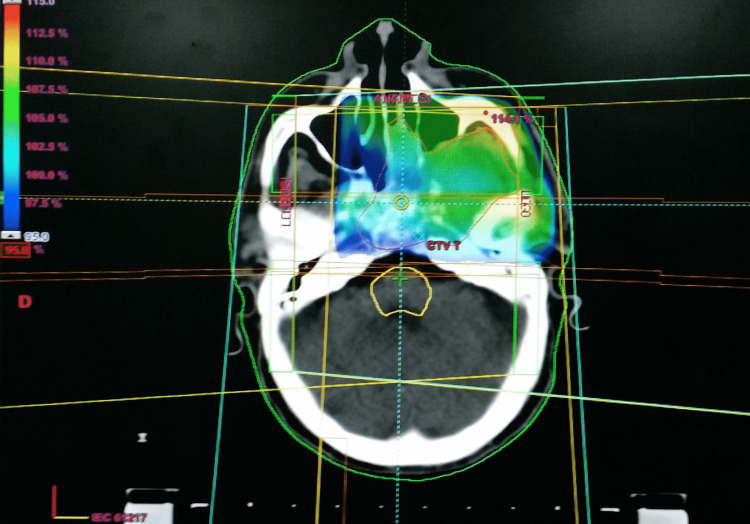
Axial view of the treatment plan and dose distribution. Intensity-modulated radiotherapy was used to deliver 70 Gy in 35 fractions along with concomitant weekly cisplatin. The color code for isodose lines is showed in the upper right hand corner.

**Figure 2:**
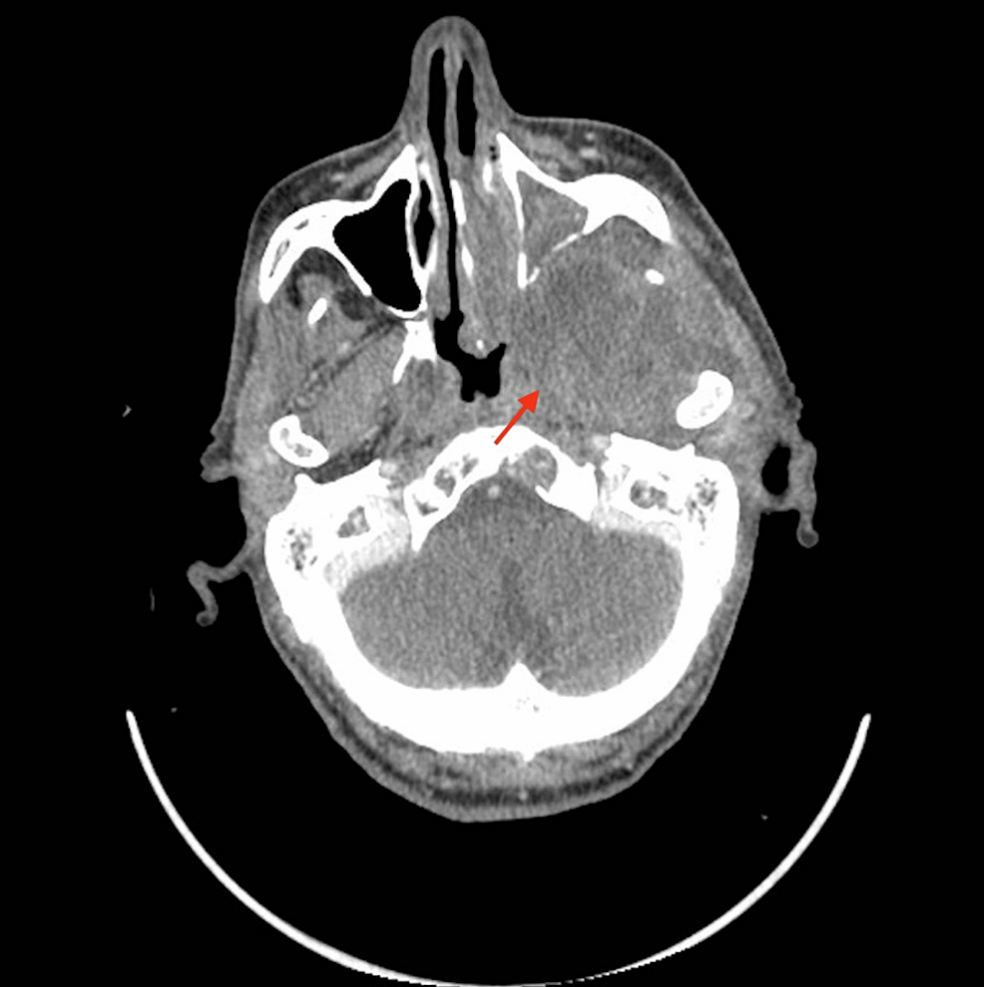
A CT scan performed six months after the end of radiation therapy showed a stable disease, with a residual lesion that extends to the infra-temporal fossa (arrow).

A 63-year-old man, a regular smoker, hypertensive with hyperuricemia and a history of acute ischemic stroke in 2000, was referred to our hospital with a six-month history of headaches, left nasal obstruction, and repeated epistaxis. The patient reported left-sided facial pain that had been present for three years ago. Physical examination confirmed trigeminal neuralgia; there was no palpable neck mass. Nasopharyngoscopy showed an exophytic, ulcerative, and easily hemorrhagic tumor on the left side of the nasopharynx. A core needle biopsy was performed. Microscopic examination showed a proliferation of carcinoma cells with clear cytoplasm and positive immunochemical staining for pan-cytokeratin (Dako, Agilent, Santa Clara, CA; monoclonal; AE 1, AE 3), epithelial membrane antigen (EMA), and vimentin. Focal and weak staining for high molecular weight keratin CK5/6 was seen. The tumor cells stained negative for CD 10, S 100 protein, CK 7, CD 117, TTF1, thyroglobulin, melanoma antigen (Melan A), and prostate-specific antigen (PSA). This histological aspect suggests the diagnosis of primary salivary gland type clear cell carcinoma, not otherwise specified, of the nasopharynx. The staging procedures revealed a locally advanced nasopharyngeal tumor that erodes bony structures of the skull base with extension to the infratemporal fossa and intracranial extension. No distant metastasis was found and no primary tumor of the kidney was identified. Thus, according to the American Joint Committee on Cancer (AJCC) staging system for nasopharyngeal carcinoma (NPC), 8th edition (2017), the disease was classified as stage IV-a (T4N0M0). The Eastern Cooperative Oncology Group (ECOG) performance status was assessed as 1. Complete blood counts, liver, and renal function tests were normal. An electrocardiogram showed left ventricular hypertrophy. Echocardiography was normal with a left ventricular fraction ejection equal to 63%. The patient was treated by induction chemotherapy based on platinum and 5FU (PF) regimen consisting of infusional 5-FU at a dose of 1000 mg/m^2^/d for five days in combination with cisplatin 75 mg/m^2^ on day 1 every three weeks for three cycles, followed by radiotherapy (RT) with concurrent chemotherapy. Radiation was delivered during a seven-week period with the use of conventional fractionation (total dose to 70 Gy, 35 fractions) (Figure 1). Concurrent chemotherapy was based on weekly cisplatin at a dose of 40 mg/m^2^. Induction chemotherapy was well tolerated with the exception of alopecia and grade 1 nausea and fatigue. During concurrent chemoradiotherapy, the patient complains of stomatitis and odynophagia. After six months of follow-up, the patient reports a clinical benefit with the disappearance of nasal obstruction, headaches, and epistaxis. However, the patient experiences xerostomia as a sequel to radiation therapy. The response rate assessed according to response evaluation criteria in solid tumors 1.0 shows a durable stable disease after 6 (Figure 2) and 12 months of follow-up.

## Discussion

Clear cell carcinoma, not otherwise specified, of minor salivary glands, also known as hyalinizing clear cell carcinoma, is an unusual primary malignancy of the nasopharynx [1,3]. To the best of our knowledge, there are 13 cases reported in the literature [5]. Only eight of them have complete clinical data reported (Table 1) [3,5-11]. These tumors are microscopically characterized by a monomorphous population of cells with optically clear cytoplasm with standard hematoxylin and eosin stains [2]. Tumor cells are arranged into sheets, nests, or cords. Cytoplasmic glycogen assessed by periodic acid Schiff stain varies from marked to not evident. Tumor cells usually stain negative for mucin. Mitotic figures are rare. Tumors cells stained positive for cytokeratin, however variable results are reported regarding S100 protein, glial fibrillary acidic protein, actin, and vimentin staining [2]. At a molecular level, EWSR1 (Ewing sarcoma breakpoint region 1) rearrangement has been described as a hallmark of clear cell carcinoma of the salivary glands and may be useful for the diagnosis [7]. Differential diagnosis includes other salivary gland cancers [3] and metastatic clear cell carcinoma arising from other tissue, particularly the thyroid or the kidney [6-12]. An appropriate diagnostic workup helps to rule out metastatic lesions, and EWSR1 rearrangement may be useful to distinguish clear cell carcinoma from other salivary glands [13].

These tumors occur in patients in the 22-63-year age range with no sex predilection [9,6]. They have indolent clinical courses and the history may go back many months or even years [2-8]. They are at low risk of lymph node and distant metastasis [1-8]. 

Because of the rarity of these tumors, there are no adequate clinical trials that define the optimal therapeutic approach. Surgery is the mainstay of treatment of minor salivary gland tumors [1-4]. Data on the efficacy of chemoradiotherapy are limited [4]. There are no specific recommendations regarding the site of origin. Surgery +/- postoperative irradiation was preferred in the eight reported cases (Table 1). In the present case, the tumor was inaccessible for surgery because of the local extension of the lesion (T4) and because NPCs are not classically managed by surgery in our region. Thus, the patient was treated following locally advanced NPC recommendations with platinum-based induction chemotherapy followed by concurrent chemo-radiotherapy [4]. After three cycles of the PF regimen, the response rate shows stable disease, suggesting a lack of chemotherapy sensitivity in this histological subtype. Chemoradiotherapy does not result in a higher response rate. However, this approach has led to adequate palliation of disease-related symptoms with a favorable toxicity profile. Various systemic options including other chemotherapeutic agents (cisplatin/cyclophosphamide/doxorubicin, cisplatin/vinorelbine, carboplatin/paclitaxel, carboplatin/gemcitabine), targeted therapies (bicalutamide, trastuzumab, axitinib, sorafenib, sunitinib, dovitinib, neurotrophic tyrosine receptor kinase (NTRK) inhibitors, and lenvatinib), or immunotherapy (Pembrolizumab) have been shown active for some salivary gland malignant histologies [4]. However, none of these studies have included the hyalinizing clear cell carcinoma subtype. Nevertheless, because of the lack of other evidence-based options for these tumors, we believe that screening for targetable biomarkers such as androgen receptor, NTRK, HER 2, and TMB may benefit some patients with advanced unresectable clear cell carcinoma of the nasopharynx [4].

**Table 1:** Reported cases of salivary gland clear cell carcinoma of the nasopharynx and the present case *Stage was deducted from data provided on imaging assessment. **Incomplete surgical excision.

Case	Age	Gender	Stage*	Treatment	Follow up-outcome	Author (reference)
1	51	Female	-	Surgery radiotherapy	Recurrence after 1, 3, 6, and 12 years	Tang and Wan [8]
2	63	Female	T1N0M0	Endoscopic resection radiotherapy	12 months - no recurrence	Cheng et al. [6]
3	38	Male	T4N1M0	Surgery (R2**) radiotherapy chemotherapy	-	Ceballos Sáenz et al. [7]
4	27	Female	T1N0M0	Surgery	>2 years - no recurrence	Nakashima et al. [3]
5	48	Male	T1N0M0	Surgery + radiotherapy	9 weeks post-treatment - no residual disease	Malfitano et al. [5]
6	63	Female	T1N0M0	Endoscopic excision	1 year - no recurrence	﻿Atsushi Fukuda et al. [9]
7	22	Female	T1N0M0	Endoscopic excision + radiotherapy	3 years - no recurrence	Dosemane et al. [10]
8	62	Male	T1N0M0	Excisional biopsy	5 months - no recurrence	Chapman et al. [11]
9	63	Male	T4N0M0	Induction chemotherapy > concurrent chemoradiotherapy	12 months - stable disease	Present case

## Conclusions

Primary salivary gland clear cell carcinoma of the nasopharynx is uncommon. An appropriate workup is mandatory for a definitive diagnosis. Optimal treatment for locally advanced unresectable tumors is unclear. Further studies are needed to define the appropriate treatment strategy. A better understanding of the biology of the tumor and the identification of potential targets is warranted for new drug development.
